# 3D Creatine Kinase Imaging (CKI) for In Vivo Whole-Brain Mapping of Creatine Kinase Reaction Rates with ^31^P-Magnetization Transfer MR Fingerprinting

**DOI:** 10.21203/rs.3.rs-5271263/v2

**Published:** 2024-11-15

**Authors:** Mark Widmaier, Antonia Kaiser, Pontus Pandurevic, Song-I Lim, Andre Döring, Zhiwei Huang, Daniel Wenz, Ying Xiao, Yun Jiang, Lijing Xin

**Affiliations:** 1 CIBM Center for Biomedical Imaging, Switzerland.; 2 Animal Imaging and Technology, Ecole Polytechnique Federale de Lausanne (EPFL) Lausanne,Switzerland.; 3 Laboratory of functional and metabolic imaging, Ecole Polytechnique Federale de Lausanne (EPFL) Lausanne,Switzerland.; 4 Institute of Physics, Ecole Polytechnique Federale de Lausanne (EPFL) Lausanne,Switzerland.; 5 Department of Radiology, University of Michigan, Ann Arbor, Michigan, USA.

**Keywords:** MRF, 31P, Creatine Kinase Imaging, CKI, functional Imaging, fCKI, bioenergetics

## Abstract

The creatine kinase (CK) is a key enzyme involved in brain bioenergetics, playing a key role in brain function and the pathogenesis of neurological and psychiatric diseases, but imaging its activity noninvasively in the human brain in vivo remains a significant challenge. This study aims to advance the magnetization transfer (MT)- ^31^P magnetic resonance fingerprinting (MRF) for 3D Creatine Kinase Imaging (CKI). The method was implemented and validated on a clinical 7 Tesla MRI scanner. It enables whole-brain mapping of CK reaction rates for the first time, showing robust reproducibility for 25-minute scan sessions. CKI acquisition also provided simultaneous mapping of adenosine triphosphate and phosphocreatine concentration ratios, phosphocreatine longitudinal relaxation time, and $B_{0}$ maps. Furthermore, a functional CKI (fCKI) study demonstrated the first CK activation map in response to visual stimulation, revealing a mean 15% increase in CK rates in the visual cortex. The novel imaging modalities, CKI and fCKI, have the potential to offer new insights into brain bioenergetics both at rest and during activity.

## Introduction

Maintaining brain function requires 20% of the body’s energy. Creatine kinase is a key enzyme that catalyzes the reversible reaction between creatine and phosphocreatine (PCr), facilitating the conversion of ATP to ADP. This reversibility enables PCr to function as an energy reservoir, allowing rapid ATP buffering, regeneration, and intra-cellular energy transport through the PCr shuttle. Creatine kinase thus plays a crucial role in cellular energy buffering and transport, supporting neuronal activities. CK reaction rate indicates mitochondrial function and is regulated during functional events such as physical training in the muscle[1–3] and visual stimulation in the brain[4–6]. Creatine kinase dysfunction has been reported to play a critical role in aging[7] and the pathophysiology of various neurological diseases, including psychiatric disorders [8, 9] and neurodegenerative diseases[10–12].

^31^P-MRS/I non-invasively reveals bioenergetics in vivo by assessing the levels of energy metabolites such as ATP, PCr, and Pi. Beyond the quantification of concentrations, ^31^P-MRS can assess the chemical reaction rate of CK through magnetization transfer (MT) experiments, including saturation transfer[13–17] (ST) or inversion transfer[3, 18–24] (IT) techniques. However, ^31^P-MRS in general suffers from low sensitivity relative to ^1^H MRI due to low metabolite concentrations (e.g., PCr 0.004 M vs. water 43 M in the human brain) and a lower gyromagnetic ratio, approximately 2.5 times less than that of ^1^H. Signal averaging is commonly used to address this issue; however, this comes at cost of prolonged acquisition time. Long $T_{1}$ relaxation times in ^31^P metabolites further increases acquisition time and hinder efficient spatial sampling. Therefore, mapping CK reaction rates in the human brain remains highly challenging. So far, only one report by Bottomley et al.[25] from 1992 demonstrated a 2D CK map with a spatial resolution of 20 × 20 × 50mm^3^ using ST and Cartesian spatial encoding at 4T with a total scan time of 34 min and a repetition time (TR) of 1s.

Although ST methods have been the traditional choice due to strong MT effect, the use of saturation pulses results in excessive RF deposition, reducing the measurement efficiency and limiting their application in the human brain at ultra-high fields (UHF) due to specific absorption rate (SAR) limits. To benefit from the increased signal sensitivity at UHF, IT methods have gained attention recently their lower SAR burden[3, 26]. However, both ST and IT magnetization transfer experiments require multiple measurements, involving varying saturation or inversion times, to determine CK reaction rates, as well as T1 relaxation times and levels of PCr and ATP. Recently, a novel approach to CK kinetic measurement was undertaken by Wang et al. in rodent muscle[27] (CK-MRF) and by Widmaier et al. in the human brain[28] (MT-^31^P-MRF), adapting the magnetic resonance fingerprinting (MRF) framework[29] to phosphorus MT measurements. The MRF framework has proven to be time efficient for multi-parametric estimations. Similar to the initial application for proton MRI, a balanced steady-state free precession (bSSFP-)type of acquisition scheme was chosen in phosphorus MRF applications for its highest signal-to-noise ratio (SNR) efficiency among existing MR sequences. The MT approach, however, differs between the two studies. Wang et al. used a ST scheme, as rodent applications are not limited by SAR. Widmaier et al. employed an IT approach for the implementation at UHF with a human brain application. CK-MRF and MT-^31^P-MRF both demonstrated the potential of MRF for estimating CK reaction rates, showing improved precision in shorter acquisition times compared to reference methods, with up to a 4-fold scan time reduction in human brain applications. However, MT-^31^P-MRF has, so far, been restricted to a small region of interest (ROI) using 1D localization.

In this work, the MT-^31^P-MRF framework has been advanced to perform 3D Creatine Kinase Imaging (CKI), delivering, for the first time, full-brain CK reaction rate maps. This was achieved by a SNR efficient sequence design, with an ultra-short acquisition delay time (TE) of 0.5 ms and a bSSFP-type sequence using a spiral k-space sampling. CKI leverages a target-oriented imaging approach by selectively sampling either PCr or γ-ATP maps. In addition to CK reaction rates, the CKI acquisition scheme enables simultaneous mapping of the concentration ratio between ATP and PCr, the longitudinal relaxation time of PCr, and phosphorus-based $B_{0}$ maps at an unprecedented resolution of 7.2 × 7.2 × 20 mm^3^. Notably, the CKI application offers ease of use with a simple push-button acquisition and reconstruction process. The technique was first demonstrated with a 49:30-minute acquisition time on a clinically approved MRI scanner. Within-session reproducibility further suggests that a feasible application could be achieved in just 25 minutes. In a proof-of-concept application, CKI was applied dynamically during visual stimulation, providing the first-ever 3D CK activation clusters in the visual cortex. This CKI application introduces a novel functional imaging modality, functional Creatine Kinase Imaging (fCKI), which provides insights into the bioenergetic mechanisms underlying brain function.

## Results

### Creatine Kinase Imaging (CKI) sequence

**Fig. 1:**
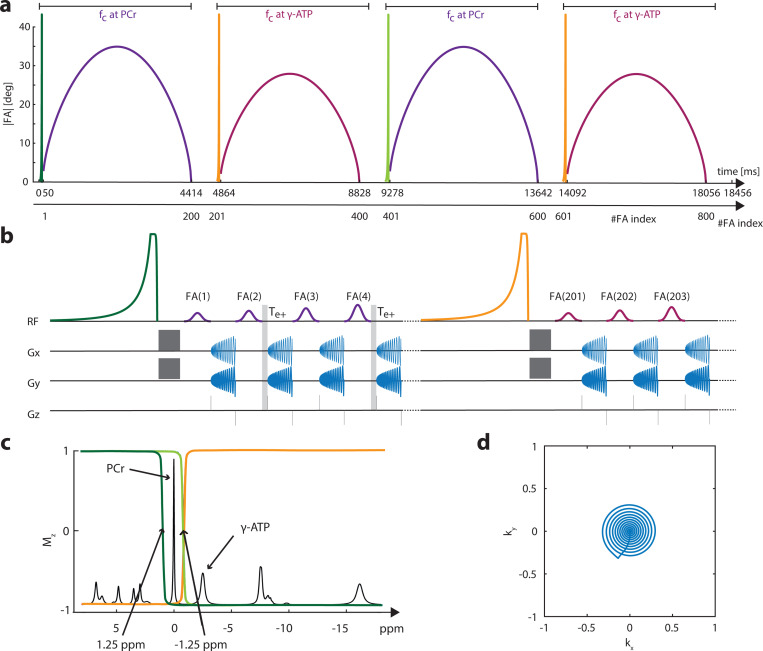
Sequence schematics: (a) The flip angle (FA) pattern with 800 FAs in total, consisting of 2 × 200 FA blocks with carrier frequencies at PCr (purple) and γ-ATP (magenta), respectively. (b) Zoom- in view of the beginning part of the sequence diagram, showing additional delay $T_{e+}$ for every second PCr acquisition. (c) An illustration of the pulse profiles of the magnetisation transfer preparation pulses, prior to the respective 200 FA blocks. The same colour scheme as shown in (a): the pulse in dark green is applied before the 1st block; the pulse in light green is applied before the 3rd block and the one in orange is applied before the 2nd and 4th blocks. (d) The spiral trajectory for one $k_{xy}$ plane with a 33% coverage.

Similar to our previous 1D MT-^31^P-MRF approach[28], a bSSFP-type sequence (Fig. 1a) is used to achieve high SNR efficiency for CKI. The gradient moment is set to be net-zero for each TR (Fig. 1b), and the FA input pattern (Fig. 1a) is applied with alternating phases of 0° and 180°. In this approach, 3D ^31^P images of PCr and γATP are acquired as inputs for a two-pool chemical exchange model to estimate the creatine kinase rate $k_{\text{CK}}$, the concentration ratio $C_{\text{r}}=\frac{M_{\text{PCr}}^{0}}{M_{\text{ATP}}^{0}}$, the longitudinal relaxation time of PCr $T_{1}^{\text{PCr}}$, and the off-resonance $\text{Δ}B_{0}$. Frequency selectivity on the metabolites of interest (PCr or γATP) was achieved by a 10 ms Gaussian pulse with a bandwidth of 170 Hz, while the frequency difference between PCr or γATP is ∼ 300 Hz. Furthermore, the signal obtained in a bSSFP-type of sequence is off-resonance dependent and exhibits a periodic pattern with alternating passbands and stopbands. The widths of these bands depend on the TR and FA. Therefore, TR and FA in the bSSFP was also optimized to minimize signal contamination to the metabolites of interest from the non targeted metabolites. A TR of 19.82 ms was chosen, with < 1% contamination in the expected FA range given by the input pattern (Supplementary Fig. S1).

### In Vivo Demonstration

**Fig. 2:**
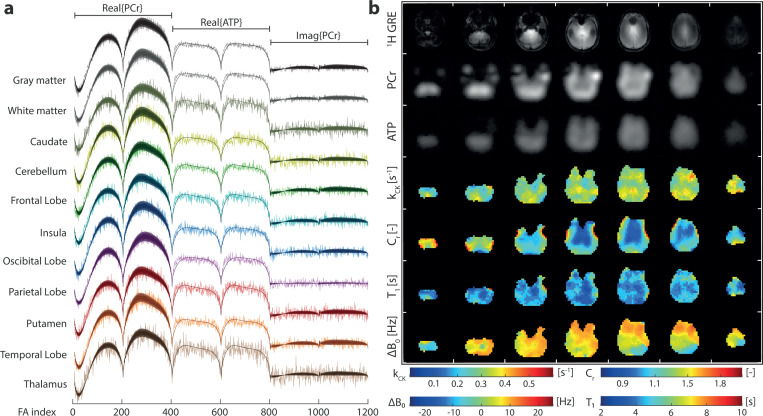
(a) Examples of measured signal evolutions and their matched dictionary entries of different ROIs. (b) In vivo brain images of the same participant including reference ^1^H GRE images, the ^31^P bSSFP-type images (sum along FA index) for PCr and ATP, the $k_{\text{CK}}$, $C_{\text{r}}$, $\text{Δ}B_{0}$ and $T_{1}^{\text{PCr}}$ maps. Colour map ranges are set according to the dictionary limits for $k_{\text{CK}}$, $\text{Δ}B_{0}$ and $T_{1}^{\text{PCr}}$. $C_{\text{r}}$ colour range limits were set to increase the visibility of gray and white matter differences.

Examples of in vivo signal evolutions and their matched dictionary entries for different region of interests (ROIs) of participant 6 are shown in Fig. 2a. The transversal maps of $k_{\text{CK}},\;C_{\text{r}},\;\text{Δ}B_{0}$, and $T_{1}^{\text{PCr}}$ with a 7.2 × 7.2 × 20 mm^3^ resolution obtained in a 49:30-minute acquisition are shown in Fig. 2b. For anatomical reference, ^1^H GRE images and the ^31^P-bSSFP-type images of PCr and ATP are shown above. Overall, the mean values of $k_{\text{CK}}$ (Table 1) are in the range of reported values (Table 2). No significant difference of $k_{\text{CK}}$ between gray and white matter tissues was found. However, Cerebellum shows lower $k_{\text{CK}}$ values relative to 3 other ROIs (Insula, Occipital and Pariental Lobe; Supplementary Information Table S6 & S8). A significant lower (*p* < 0.01) mean $C_{\text{r}}$ value in white matter than gray matter was detected (Supplementary Information Table S5). With a mean $C_{\text{r}}$ of 0.98, 1.00, 1.03 and 1.12, the Thalamus, Caudate, Putamen and Insula showed significant lower concentration ratio compared to other ROIs. On the other hand, Cerebellum and Temporal Lobe showed significant higher concentration ratios, with a $C_{\text{r}}$ of 1.42 and 1.36 respectively, compared to other ROIs. $T_{1}$ maps and values show no significant difference between gray and white matter, yet some significant differences between ROIs are found (Supplementary Information Table S5). Note that no interaction between sex and tissue or ROI was found with the sample size of this study(Supplementary Table S7 & S8). The $\text{Δ}B_{0}$ maps show that most areas are within a ±10 Hz range. Towards the frontal lobe, the values of $\text{Δ}B_{0}$ deviate as expected due to locally high susceptibility effects.

### Intrasession Reproducibility

To assess the reproducibility of the measurements and the possibility of reducing scan time by using fewer averages, the full dataset was split into two subsamples, each containing 16 averages, to calculate the intrasession coefficient of variation (CV). Table 1 shows the CV values for $k_{\text{CK}}$, $C_{\text{r}}$, and $T_{1}^{\text{PCr}}$. The mean CV across all participants and ROIs did not exceed 10.2%, 3.5%, and 10.7% for $k_{\text{CK}}$, $C_{\text{r}}$, and $T_{1}^{\text{PCr}}$, respectively. Excellent intrasession reproducibility was achieved with half of the measurement time in gray and white matter, with mean CVs below 4.9%, 2.1%, and 4.0% for $k_{\text{CK}}$, $C_{\text{r}}$, and $T_{1}^{\text{PCr}}$, respectively. In addition, noise robustness was evaluated through Monte Carlo simulations, showing that standard deviations of matching errors of $k_{\text{CK}}$, $C_{\text{r}}$, and $T_{1}^{\text{PCr}}$ maintained respectively below 14%, 5%, 16.5% when SNR > 8 dB, highlighting the reliability of the method (Supplementary Fig. S3).

### Functional Creatine Kinase Imaging

**Fig. 4:**
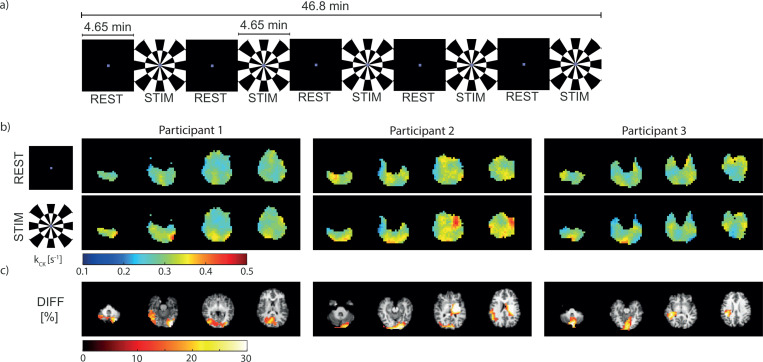
(a) The visual stimulation paradigm consists of 5 interleaved blocks of rest (REST) and stimulation (STIM). Each block takes 4.65 min, resulting in a total duration of 46.8 min. (b) In vivo brain $k_{\text{CK}}$ maps estimated from the averaged REST and STIM blocks, respectively, and (c) the percentage difference change (DIFF) from REST to STIM. Clusters (size ≥ 20) with a change ≥ 10% are displayed. The DIFF map is overlaid on a coregistered $T_{1}$-weighted ^1^H-MR image.

A functional study using dynamic CKI is demonstrated, as an exemplary application of CKI. Fig. 4a shows the visual stimulation paradigm applied in fCKI. An increase in $k_{\text{CK}}$ is visually notable in the visual cortex of the brain between REST and STIM (Fig. 4b). Segmentation revealed for each participant a mean absolute $k_{\text{CK}}$ increase of 0.04 s^−1^ in the occipital lobe, with a mean relative increase of 15% (p=0.07). This finding is in line with the percentage difference map (DIFF) in Fig. 4c. Activation clusters (size ≥ 20) with a percentage difference in $k_{\text{CK}}$ between STIM and REST blocks (DIFF) ≥ 10% are primarily located in the posterior part of the brain, predominately within the visual cortex.

## Discussion

The CKI sequence achieved excellent performance in vivo, allowing for the first time the measurement of whole-brain maps of $k_{\text{CK}}$, $C_{\text{r}}$, $\text{Δ}B_{0}$, and $T_{1}^{\text{PCr}}$ with the highest resolution achieved so far of 7.2 × 7.2 × 20mm^3^. The obtained values in different ROIs are consistent with previously reported values (Table 2). The reproducibility of CKI was evaluated through within-session variability analyses. Half of the scanning data (16 averages, 25 min), shows mean CVs below 11% for all parameters and ROIs, indicating consistent performance.

In this proof-of-concept study, the method was embedded in a functional task, demonstrating fCKI as a potential new functional imaging modality that offers a means to inspect brain activity from a bioenergetic perspective. In a visual stimulation task, activation clusters were detected predominately in the visual cortex. For the first time, single-subject spatial information on CK functional activation is provided. The detected 15% mean increase of $k_{\text{CK}}$ is in line with a previous single voxel study which reported 17.5% increase during visual stimulation of similar length (average changes over both cycles)[5]. However, this increase is lower than the first report (34%) from Chen et al.[5] using a LED goggle flashing at 8Hz, and higher than a recent report[6] (5%) using a ^31^P-MT CSI sequence. Note that all previous results were based on the group analysis. In a future study, more participants are needed to evaluate the potential of fCKI comprehensively.

The FA pattern of simple sinusoids in combination with magnetization preparation pulses has proven to be effective for the sensitivity of parameter estimation in prior MRF frameworks[28, 36]. Incorporating IR and MT pulses increased the sensitivity to $T_{1}$ and $k_{\text{CK}}$, similar to our previous work[28]. IR for ${\text{T}}_{1}$ sensitivity was applied only to PCr due to its higher SNR relative to ATP, and the ATP acquisition pattern is repeated twice to boost SNR. For the same reason $T_{e}$ was only varied in the PCr acquisition, to provided additional sensitivity to $\text{Δ}B_{0}$. Although the feasibility of this pattern is demonstrated, it should not be considered as optimal. Future work could increase parameter sensitivity, matching robustness, and SNR by optimizing the design of the acquisition scheme[37–39].

At ultra-high fields, $B_{0}$ inhomogeneities are a common obstacle for MRI. As bSSFP-type acquisitions are especially sensitive to changes in $B_{0}$, incorporating $\text{Δ}B_{0}$ in the estimation is necessary. This approach accounts for pattern changes caused by the shift in the bSSFP frequency response profile and frequency-selective pulse profile. However, inhomogeneities due to susceptibility effects in the frontal area remain challenging, as in this region signals may fall into stop bands of the bSSFP frequency response profile leading to significant signal loss. This issue can be addressed by local shimming on the affected area, trading off $B_{0}$ homogeneities in other regions, or by using additional local shim coils to mitigate the effect.

$B_{1}$ inhomogeneity may present as a challenge in MRF[29, 40]. Considering the similar Larmor frequency between ^1^H at 3T and ^31^P at 7T, and the use of a birdcage volume coil for transmission, a homogeneous transmit field is assumed in this proof of concept study, similar to the approach in the initial demonstration in ^1^H MRF by Ma et al.[29]. However, incorporating $B_{1}$ maps could significantly improve the robustness of parameter estimation (Supplementary Fig. S2). Although the acquisition of experimentally measured $B_{1}$ maps could be time-consuming, our recent work indicates that ^31^P whole-brain $B_{1}$ maps are feasible in less than 15 minutes, making it a practical option [41]. An alternative solution could be found by optimizing the FA pattern to be less sensitive to $B_{1}$ or include $B_{1}$ as a parameter to be estimated[40]. Incorporating $B_{1}$ in the fitting is expanding the computational complexity, as the dictionary size grows exponentially with the number of parameters to fit.

For the same reason in the current approach, $T_{2}$of PCr and ATP, and $T_{1}^{\text{ATP}}$ are fixed to literature values. Therefore, the matching time and computational load in dictionary generation is reduced, similar to prior work[27, 28]. Simulations showed that the $k_{\text{CK}}$ estimation is robust to a bias between the assumption and ground truth values. The matching error stays below 13% for ±25% alteration of the fixed assumption for each of the three parameters (Supplementary Fig. S2). As mentioned in earlier publications [27, 28], no studies have investigated changes in $T_{2}$ of PCr and ATP in the brain upon pathological conditions. However, there are reports of alterations in $T_{2}$ of ^1^H metabolites in ischemic tissues and during brain development[42, 43], which leaves the debate open if phosphorus $T_{2}$ values might also facing alterations in different health and pathological conditions. If the computational challenges can be addressed in the future, incorporating $T_{2}$ into the matching process is expected to enhance the accuracy of parameter estimations.

The use of a two-pool (PCr-ATP) chemical exchange model simplifies the problem further. As shown in previous ^31^P-MRF studies[27, 28], the interaction with the inorganic phosphate pool over the ATP synthesis was mitigated. For CKI a overestimation of 6% can be expected within the in vivo range of the ATP synthase exchange rate (Supplementary Fig. S8). Including the ATPase exchange rate in future analyses could refine the model further. To do so, the input signals might need to be extended (e.g. Pi maps), potentially lengthening the acquisition time. Incorporating all parameters mentioned above will increase the complexity of the model and more sophisticated matching approaches are needed to avoid a computational overload. This could potentially be addressed by using the nested iteration interpolation method (NIIM)[28] or AI-based approaches.

The CKI is a user-friendly method with a push-button acquisition. The reconstruction and matching procedures are achieved fully automated. The excellent robustness of the method, coupled with the easy application, underscores the potential for its use in clinical studies. Moreover, the method is not restricted to the brain; with adjustments to the readout trajectory and pattern to accommodate different tissue properties, it can be applied to other organs.

In conclusion, CKI provides a novel imaging method for whole-brain $k_{\text{CK}}$ mapping. Offering high SNR efficiency and easy applicability with push-button acquisition and reconstruction, CKI can be used for future clinical and research applications. Applied along with a functional task, fCKI provides a new functional imaging modality. The $k_{\text{CK}}$ activation mapping allows unprecedented access to information on brain bioenergetics underlying brain function. CKI and fCKI have the potential to enhance our understanding of brain function and neurological dysfunctions, including neurodegenerative and psychiatric disorders.

## Methods

### ^31^P-MRF Sequence Design

The FA pattern shown in Fig. 1a comprises 800 radio frequency (RF) pulses, organized into 4 sinusoids of 200 FAs each ($A\cdot \text{sin}{\left(\pi /200\cdot x\right)}^{\mathrm{0.6}}$). Sinusoids 1 and 3 have a maximal FA of *A* = 35°, while sinusoids 2 and 4 have a maximal FA of *A* = 25°. The maximum amplitude of the sinusoids was chosen to maximize the SNR (Supplementary Fig. S1b). Preceding each sinusoid, a 40 ms asymmetric inversion pulse[44] prepares the longitudinal magnetization, followed by a 2 ms delay and a 8 ms crusher gradient to spoil the transverse magnetization (Fig. 1b). The carrier frequency (at the center of the transition band) is set at +1.25 ppm for the first and −1.25 ppm for the second to the fourth preparation pulses (Fig. 1c). Inversion profiles for the first and third preparation pulses are opposite to those of the second and fourth. After the last FA of a sinusoid, a 400 ms pause is added to allow partial relaxation of the longitudinal magnetization. The RF carrier frequency of excitation pulses in the first and third sinusoids is set at 0 ppm for PCr, while in the second and fourth sinusoids, it is set at −2.52 ppm for γ-ATP. Frequency selective excitation is achieved with a 10 ms Gaussian pulse (FWHM=170 Hz). The TR alternates for PCr acquisition between $T_{R,1}=19.82$ ms and $T_{R,2}=T_{R,1}+T_{e+}=23.82$ ms, while for ATP acquisition, it remains fixed at $T_{R,1}$. The delay time between the end of the RF pulse and the start of the acquisition is $T_{e}=410$ *μ*s, including the spatial encoding gradient in $k_{z}$. Every second PCr acquisition includes an additional delay of $T_{e+}=4$ ms before $T_{e}$. The readout trajectory in $k_{xy}$ is a 8.61 ms (861 points) non-uniform spiral with 33% $k_{xy}$ coverage (Fig. 1d). This readout is followed by a 920 *μ*s delay, including refocusing in $k_{xy}$ and $k_{z}$. Five repetitions of the whole FA pattern are needed to measure the 5 central $k_{z}$ planes from the overall 11 $k_{z}$ planes. The outer 3 $k_{z}$ planes on each side are zero-filled. In total, 1:33 min is needed to acquire one 3D volume.

### Dictionary Creation and Parameter Matching

A dictionary was created using the Bloch-McConnell equations. A single isochromat per voxel and RF excitation with the nominal FA is assumed. 4 parameters were estimated through MR fingerprinting signal matching: the CK reaction rate $k_{\text{CK}}$, the concentration ratio $C_{\text{r}}=M_{PCr}^{0}/M_{ATP}^{0}$, the longitudinal relaxation time of PCr $T_{1}^{\text{PCr}}$, and the off-resonance $\text{Δ}B_{0}$. Parameter ranges are listed in the Supplementary information 4. To simplify the matching problem, another 3 parameters were fixed to literature values[28, 35]: $T_{1}^{\text{ATP}}=1\;\text{s},T_{2}^{\text{PCr}}=135\;\text{ms}$, and $T_{2}^{\text{ATP}}=25\;\text{ms}$. Measured MRF signal evolutions and dictionary entries were processed equally before matching. Individual PCr signal evolutions were phased according to the mean phase of all odd FAs (the transient with no $T_{e+}$). Individual ATP signal evolutions were phased according to the mean phase of all ATP signal evolutions. The real (400 points) and complex (400 points) parts of the PCr signal evolution and the real part of the ATP signal evolution (400 points) were concatenated, resulting in a signal evolution with 1200 points in total. Matching was performed on the L2-norm of signals by finding the maximum inner product of the dictionary entries and measured MRF signal evolutions. Reconstruction and matching was completed in approximately 1 hour on a dedicated server (Intel Xeon Silver 4216).

### In Vivo Validation (CKI)

Six healthy participants (3 female; 3 male; aged 26.5 ± 3.9 years old), who provided written informed consent, were included in the study. MR experiments were conducted on a Siemens Terra X 7T/80 cm MR scanner (Siemens Healthineers, Erlangen, Germany). Participants were scanned using a double-tuned (Tx/Rx) birdcage coil along with a 32 channel-Rx phased array ^31^P coil (RAPID Biomedical, Rimpar, Germany). Following a localizer image, a shim volume covering the whole brain was placed, and 3D-volume shim (Siemens: GRE brain method) with intermittent frequency adjustment was applied. The mean and SD of the ^1^H linewidths in the shim area provided by Siemens frequency adjustment was 30.1 ± 2.3 Hz. Finally, the ^31^P main frequency was adjusted in the same shim volume. The MRF sequence was acquired with 32 averages, leading to a total scan time of 49:30 min including one dummy cycle. The field-of-view (FOV) was set to 230 × 230 × 220 mm^3^ centered at the isocenter. The matrix size of the reconstructed images was 32 × 32 × 11. Reference ^1^H GRE images were measured before and after the MRF protocol in the same FOV (matrix size = 128 × 128 × 11, $T_{E}=3.37$ ms, $T_{R}=15$ ms, total acquisition time of 26 sec). After the ^31^P protocol, the coil was changed to a ^1^H 32 channel Nova coil (Nova Medical, Inc. MA, USA) to acquire an anatomical MP2RAGE[45] image (4:30 min, 1 mm^3^ isotropic, FOV 230 × 230 × 224 mm^1^, TI1/TI2=870/2700 ms TE/TR=2.88/6000 ms, FA1/FA2=4°/5°) for tissue and ROI segmentation.

### Functional Creatine Kinase imaging (fCKI)

Three healthy participants (3 female; 30±2 years old), who provided written informed consent, participated in a functional study. The scan protocol of the CKI was adapted to 30 averages (46:30 min), to match the total duration of the paradigm. The paradigm is depicted in Fig. 4a and consists of alternating blocks of REST (4.65 min, 3 averages) and STIM with flashing checkerboard stimuli[46] (4.65 min, 3 averages). The participants viewed the stimuli through a mirror on a back-projection screen. Visual stimuli were full-field checkerboards, contrast reversing at 8 Hz (100% contrast). In the rest condition, a black screen with a colored fixation dot was used. In both conditions (REST and STIM), the fixation dot changed color for each average (total of 30). The participant was asked to respond to the color change with a button press (summing up all button press events = response count). The acquisition, the protocol, and the setup remained as described above (In Vivo Validation).

### Reconstruction

Reconstruction and matching were performed in MATLAB (Mathworks, Natick, Massachusetts, USA). The MRF data acquired from the scanner had a tensor size of 861 × 5 × 800 × 32 × 32, where the dimensions correspond to the spiral readout (first), the $k_{z}$ index (second), the FA index (third), the coil index (fourth), and averages (fifth). Reconstruction of the images consisted of three steps prior to matching:

1. Transformation into image domain: First, a 1D Fourier transformation was applied along the slice dimension. The density compensation function (DCF) was calculated based on the Voronoi diagram [47–49]. Before regridding, the DCF was multiplied by a half-periodic Hanning filter to reduce the influence of high-frequency components on the image. Regridding was performed using a Kaiser-Bessel kernel[50, 51] to transform the data into Cartesian k-space. Lastly, a 2D-FFT transformed the k-space data into the image domain. Images were reconstructed separately for each coil, FA index, and the $k_{z}$ index.
2. Coil Combination: Whitened singular value decomposition (WSVD)[52, 53] was used to combine the data in each voxel along the coil dimension, using the FA index dimension as a temporal dimension. The noise data used to estimate the covariance matrix was provided by the reconstruction of the interleaved subtraction of the raw data along the averages.
3. Denoising: MP-PCA[54, 55] denoising was applied on the image domain data. Therefore, the data was transformed into a two dimensional matrix by concatenated the spatial dimensions and concatenating the real and imaginary part of the signal along the FA dimension. After denoising the data was transformed back to the initial dimensions.

### In Vivo Data Analyses

The ^31^P anatomical images shown in this work from PCr and ATP are bSSFP-type magnitude images extracted from the MRF acquisition. Therefore, the signal intensity is averaged over all FA indices with the RF carrier frequency at PCr for PCr images and at ATP for ATP images. The colour bars of $k_{\text{CK}},\;\text{Δ}B_{0}$, and $T_{1}^{\text{PCr}}$ are set according to the minimum and maximum of the dictionary boundaries. For $C_{\text{r}}$, the upper limit of the colour bar was set to 2 instead of the dictionary upper bound of 6, to increase the visibility of contrast between gray and white matter.

The SNR is evaluated using the *snr* function of MATLAB. This function estimates the SNR in dB as the ratio between the signal power and noise power. The matched dictionary entry and the residual of the matched and the measured signal, used as noise approximation, are inputs for the function.

To investigate the changes in $k_{\text{CK}}$ during visual stimulation (fCKI), the percentage difference between the $k_{\text{CK}}$ maps of rest and stimulation were calculated. $k_{\text{CK}}$ maps were estimated by fixing $T_{1}$ of PCr to 4.5 sec (closest dictionary value to the mean $T_{1}$ value of gray and white matter, see Table 1) and setting $B_{0}$ and $C_{\text{r}}$ to the estimate of the combined dataset of rest and stimulation during matching. Clusters (size ≥ 20) of a percentage change ≥ 10% were considered.

### Anatomical Image segmentation

All ^1^H MR image processing and analysis were performed using FSL version 6.0.7.8[56]. Brain extraction was performed on the $T_{1}$-weighted images using the Brain Extraction Tool (BET) in FSL and manually on the reference ^1^H-GRE images using an in-house MATLAB script. BET was applied with a fractional intensity threshold of 0.5 to ensure optimal removal of non-brain tissue.

Tissue segmentation of the brain-extracted $T_{1}$-weighted images into gray and white matter was conducted using FMRIB’s Automated Segmentation Tool (FAST) in FSL. This process involved specifying the input image type as $T_{1}$-weighted, requesting three tissue-type segmentation, and applying bias field correction.

To coregister the $T_{1}$-weighted image and its segmented images to the reference ^1^H-GRE image, FMRIB’s Linear Image Registration Tool[57] (FLIRT) was employed. The $T_{1}$-weighted image was registered to the ^1^H-GRE image using six degrees of freedom (DOF) rigid body transformation. The resulting transformation matrix was then applied to the gray matter, white matter, and CSF segmented images to achieve alignment with the ^1^H-GRE.

Additionally, ROI segmentation (ATLAS 152MNI) was performed. The 152MNI ATLAS $T_{1}$-weighted image was coregistered to reference ^1^H-GRE image. Therefore, the 152MNI ATLAS $T_{1}$-weighted image was first coregistered to the participant specific $T_{1}$-weighted image with an affine registration using 12 DOFs. The participant specific $T_{1}$-weighted image was coregistered to the reference ^1^H-GRE image as described above. Nine secondary image transformations gave the region-wise segmentation (taken from the 152MNI atlas) of Caudate, Cerebellum, Frontal Lobe, Insula, Occipital Lobe, Parietal Lobe, Putamen, Temporal Lobe and Thalamus. The alignment between the registered and segmented images, and the quantitative maps from CKI were assessed with visual inspection.

### Statistical Analyses

Two-way analysis of variance (ANOVA) was performed to test for interactions between sex and ROI as well as sex and tissue type for $k_{\text{CK}}$, $C_{\text{r}}$ and $T_{1}$. Regional differences were investigated with a one-way ANOVA test, followed by a Tukey’s post hoc test to compare all pairs of VOIs. Tissue type (grey vs white matter) differences were tested by paired t-test. These tests were performed in the GraphPad Prism 5.0 (GraphPad Software, Inc., San Diego, CA). The intrasession reproducibility was assessed by splitting the full dataset (32 averages) of each participant into two subsamples of 16 averages. Reconstruction and matching were performed independently on each subsample. The CV was calculated over the two subsamples, with

(1) $$\text{CV}=\frac{\sqrt{\text{E}\left[{\left(X-\text{E}[X]\right)}^{2}\right]}}{\text{E}[X]},$$

where E[X] is the expected value.

## Supplementary Material

Supplement 1

## Figures and Tables

**Fig. 3:**
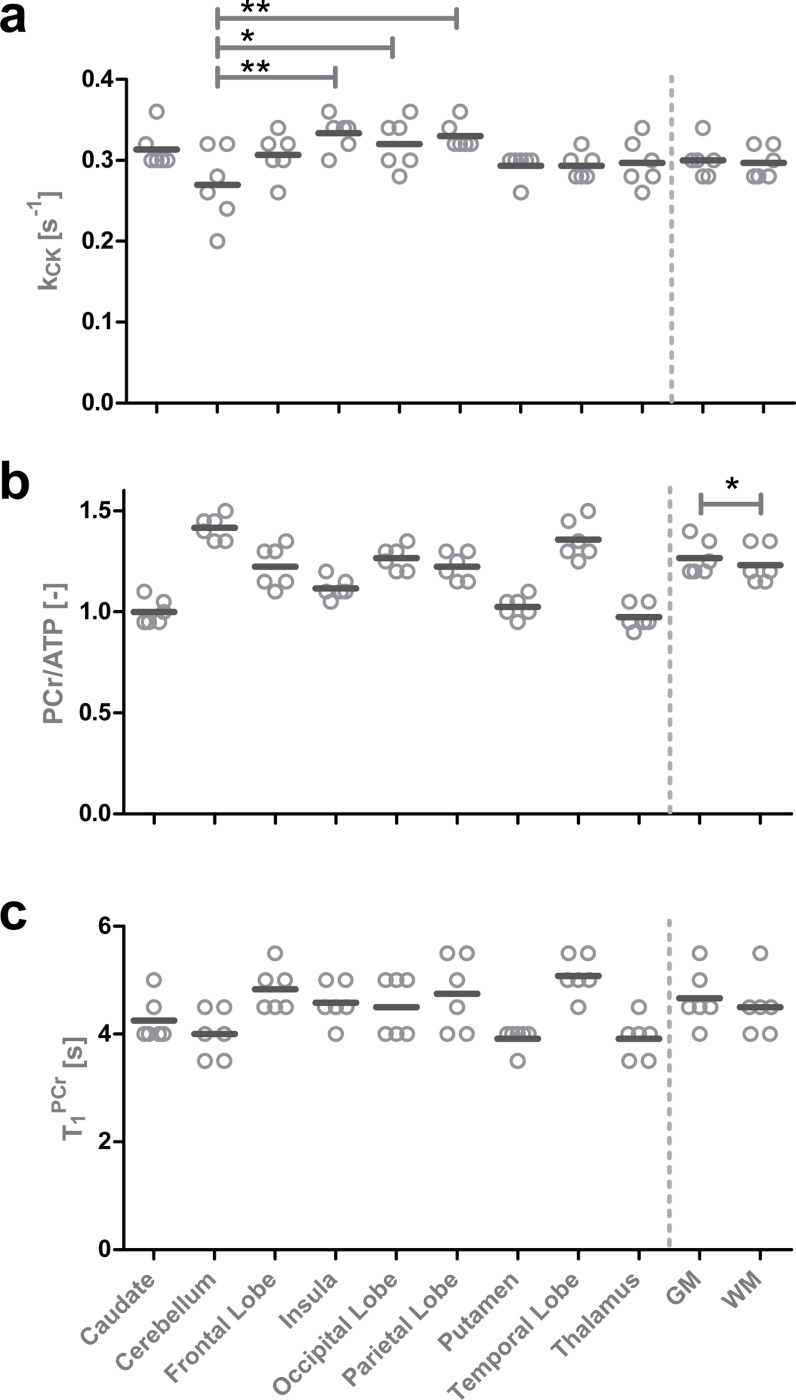
Individual and mean values of $k_{\text{CK}}$, $C_{\text{r}}$ and $T_{1}^{\text{PCr}}$ for different ROIs, and gray and white matter. Significant differences are marked if applicable for tissue difference (gray vs white matter), and also for ROI differences in $k_{\text{CK}}$ (* *p* < 0.05; ** *p* < 0.01). Significant ROI differences in $C_{\text{r}}$ and $T_{1}^{\text{PCr}}$ can be found in the Supplementary Table S5.

**Table 1:** Mean and standard deviation (SD) values of $k_{\text{CK}}$, $C_{\text{r}}$, and $T_{1}^{\text{PCr}}$ over all participants for different ROIs according to the MNI152 atlas[30, 31]. The within-session Coefficient of Variation (CV) is derived from parameter values estimated using half of the sampled data.

	$k_{\text{CK}}$ [s^−1^]		$C_{\text{r}}$ [−]		$T_{1}^{\text{PCr}}$ [s]	
ROI	Mean ± SD	CV [%]*	Mean ± SD	CV [%]*	Mean ± SD	CV [%]*
Caudate	0.31 ± 0.02	10.2 ± 9.1	1.00 ± 0.06	2.1 ± 3.6	4.25 ± 0.38	10.7 ± 6.9
Cerebellum	0.27 ± 0.04	4.2 ± 4.5	1.42 ± 0.06	2.0 ± 2.1	4.00 ± 0.41	5.4 ± 6.0
Frontal Lobe	0.31 ± 0.02	4.3 ± 3.3	1.23 ± 0.09	3.5 ± 3.2	4.83 ± 0.37	3.6 ± 5.4
Insula	0.33 ± 0.03	3.8 ± 1.7	1.12 ± 0.05	3.4 ± 1.9	4.58 ± 0.34	6.0 ± 4.6
Occipital Lobe	0.32 ± 0.03	6.7 ± 3.5	1.27 ± 0.06	1.0 ± 1.4	4.50 ± 0.50	6.7 ± 6.0
Parietal Lobe	0.33 ± 0.02	7.1 ± 6.0	1.23 ± 0.06	1.3 ± 1.8	4.75 ± 0.63	5.1 ± 5.3
Putamen	0.29 ± 0.01	3.9 ± 3.1	1.03 ± 0.05	1.2 ± 1.7	3.92 ± 0.19	5.3 ± 5.6
Temporal Lobe	0.29 ± 0.01	4.0 ± 3.3	1.36 ± 0.09	1.9 ± 2.1	5.08 ± 0.34	3.1 ± 3.2
Thalamus	0.30 ± 0.03	7.5 ± 4.8	0.98 ± 0.06	2.2 ± 2.6	3.92 ± 0.34	10.4 ± 3.5

Gray matter	0.30 ± 0.02	4.1 ± 4.6	1.27 ± 0.08	2.1 ± 1.6	4.67 ± 0.47	4.0 ± 4.1
White matter	0.30 ± 0.02	4.9 ± 3.9	1.23 ± 0.08	0.4 ± 0.9	4.50 ± 0.50	3.7 ± 3.8

* CV: intrassesion reproducibility of half sample size

**Table 2:** Summary of reported Creatine Kinase reaction rates ($k_{\text{CK}}$) in the Human Brain.

Field Strength	Method	Localization	Brain Region	$k_{\text{CK}}$ [s^−1^]
1.6T	ST	1D	Gray matter	0.30 ± 0.04 [32]
1.6T	ST	1D	White matter	0.16 ± 0.02 [32]
3T	ST	SVS	Corpus Callosum	0.32 ± 0.08[33]
4T	ST	2D-CSI	Brain Slice	0.42 ± 0.16 [25]
4T	ST	SVS	Occipital Lobe	0.56 ± 0.19 [4]
4T	ST	UL	Frontal Lobe	0.29 ± 0.02 [34]
4.7T	ST	UL	Monkey Brain	0.32 ± 0.02[19]
4.7T	IT	UL	Monkey Brain	0.29 ± 0.02[19]
7T	ST	UL	Occipital Lobe	0.24 ± 0.03[35]
7T	ST	UL	Occipital Lobe	0.30 ± 0.04 [14]
7T	ST	SVS	Occipital Lobe	0.35 ± 0.04 [5]
7T	IT	UL	Posterior Cortex	0.38 ± 0.02 [26]
7T	ST	3D-CSI	Occipital Lobe	0.38 ± 0.02 [6]
7T	MRF	1D	Occipital Lobe	0.36 ± 0.04 [28]
7T	CKI	3D-SP	Gray matter	0.30 ± 0.02*
7T	CKI	3D-SP	White matter	0.30 ± 0.02*

ST: Saturation Transfer; IT: Inversion Transfer; MRF: MR Fingerprinting; CKI: Creatine Kinase Imaging; UL: Unlocalized; SVS: Single Voxel Spectroscopy; CSI: Chemical Shift Imaging (with Cartesian spatial encoding); SP: ^31^P imaging with SPiral spatial encoding. These values were all acquired in the human brain, unless mentioned otherwise.

* (Values from this work)

## Data Availability

To make the application more accessible, our sequence is available for Siemens XA60 via c2p. The gradient readout can be easily changed using an external text file, and the sequence can be adapted to other body parts, nuclei, or other readout trajectories. The reconstruction pipeline with the look-up table estimation is freely available on GitHub (https://github.com/MRSEPFL/CKI).
