# Supplementary material for: 3D Creatine Kinase Imaging (CKI) for In Vivo Whole-Brain Mapping of Creatine Kinase Reaction Rates with 31P-Magnetization Transfer MR Fingerprinting
Source: Res Sq. 2024 Nov 15:rs.3.rs-5271263. Originally published 2024 Oct 16. Preprint. [Version 2] doi: 10.21203/rs.3.rs-5271263/v2 (PMC11527232; doi:10.21203/rs.3.rs-5271263/v2)
Supplement: Supplement 1 [file NIHPPRS5271263V2-supplement-1.pdf]

Supplementary information 1: TR Optimization

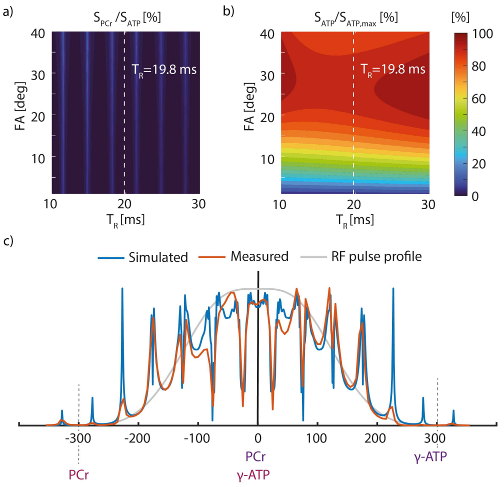

Fig. S1 TR optimization: (a) Simulated PCr signal intensity as a percentage of the ATP signal intensity, with the carrier frequency of the 10ms Gaussian pulse set at the ATP resonance frequency, is analyzed as a function of TR and FA. The white dashed line marks the TR=19.8 ms, which was chosen in this setup to accommodate sufficient time for the Gaussian pulse of 10 ms and the spatial encoding. (b) Simulated ATP signal intensity, normalized to its maximum value, as a function of TR and FA. A maximum is found at approximately 25° for TR=19.82, which was chosen as the maximal FA of the ATP sinosoids. (c) The excitation profile of the 10 ms Gaussian pulse when applied in a gradient echo imaging sequence (grey) and in a bSSFP sequence with a FA of 35° (blue and orange). Simulated (blue) and measured (orange) profiles in a phantom. The carrier frequency is set at PCr (purple label) or γ-ATP (pink label) , and they are about 300Hz apart.

Despite the sufficiency of the 10 ms Gaussian pulse for achieving frequency selectivity in gradient spoiled sequences, a bSSFP-type sequence necessitates the consideration of pass- and stop-bands[58]. To assess signal contamination from PCr during ATP excitation due to low FA excitation in stop-bands, simulations and phantom measurements were conducted. For simplicity, a bSSFP sequence with constant FA was used. PCr and ATP signals were simulated using the Bloch McConnell (BMC) equations (parameter assumptions: $T_1^{\mathrm{PCr}} = 7\,\mathrm{s}$, $T_1^{\mathrm{ATP}} = 0.85\,\mathrm{s}$, $T_2^{\mathrm{PCr}} = 135\,\mathrm{ms}$, $T_2^{\mathrm{ATP}} = 25\,\mathrm{ms}$, $C_\mathrm{r} = 1.5$, $k_{\mathrm{CK}} = 0.35\,\mathrm{s}^{-1}$). The FA varied between 5° and 40° (1° /step), and T_R varied between 10 and 30 ms (0.1 ms/step). To evaluate the signal contamination from PCr on ATP when ATP is excited, we calculated the signal ratio of PCr/ATP for each TR-FA pair (Fig. S1a). The frequency profile of the bSSFP sequence was simulated with a FA of 35° (the maximum FA of the input pattern) by varying the resonance frequency in the range of −355 Hz to 355 Hz (1 Hz/step) relative to the carrier frequency of the RF pulse. This frequency profile is validated by experiments in a phantom containing 50 mM of Pi solution, and the relaxation parameters of Pi were measured with inversion recovery and multi-TE methods ($T_1 = 3.2\,\mathrm{s}$, $T_2 = 460\,\mathrm{ms}$). Phantom experiments with a bSSFP sequence with an FA of 35° (200 repetitions) and a spiral spatial encoding, was conducted. The carrier frequency of the RF pulse was varied in

the range of $-355\,\text{Hz}$ to $355\,\text{Hz}$ in a $5\,\text{Hz}$ step, and the signal of the last repetition to ensure steady state was plotted over the frequency range (Fig. S1c). The effect of signal contamination due to low FA stop-band excitation is shown in Fig. S1a. A periodic behavior is seen determined weather PCr is falling on a stop band (strong signal contribution) or pass band (low signal contribution). Note that the effect is inverse to the nomenclature (stop/pass band) because of the bSSFP specific low FA excitation[58]. We chose a TR of 19.82 ms to minimize the signal contamination ($< 1\%$) in the expected FA range according to the FA input pattern (Fig 1a) and to have a sufficient TR to accommodate the RF pulse and the spatial encoding. If PCr falls into a stop-band, up to 25% of the signal is expected to be contributed by PCr when measuring ATP. Fig. S1c shows the simulated and measured excitation profile of PCr or ATP when the carrier frequency is set on ATP or PCr, respectively.

Supplementary information 2: The effect of biased assumption on parameter estimation

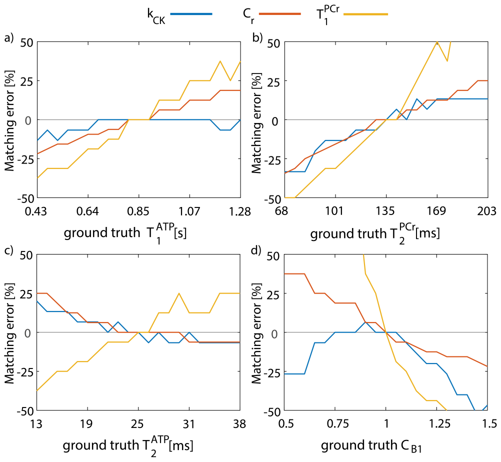

Fig. S2 Simulation results of matching errors caused by the deviation of the ground truth values from their fixed assumption for dictionary creation. Matching is displayed for free parameters: k_{CK}, C_{r} and T_1^{PCr}. No matching error was detected for B_0 and thus not displayed.

In the MRF framework, it is a common practice to reduce the complexity of the estimation process by fixing certain parameters to literature values or reasonable assumptions. This is especially the case for more complex models, including chemical exchange rates. For the proposed 3D-^{31}P-MRF approach, three parameters were fixed according to literature values, and the nominal FA is assumed over the whole FOV as B_1 field distribution is rather homogeneous at the frequency of ^{31}P. MRF signal evolutions were simulated with varying assumptions for one of the four parameters (T_1^{ATP}, T_2^{PCr}, T_2^{ATP}, B_1 scaling factor C_{B1}). The remaining parameters were fixed at the literature value. The assumptions of parameters were varied by $\pm 50\%$ relative to their fixed ground-truth values in the dictionary. Other parameters were set to $k_{\mathrm{CK}} = 0.3\,\mathrm{s}^{-1}$, $C_{\mathrm{r}} = 1.5$, $T_1^{\mathrm{PCr}} = 6\,\mathrm{s}$, and $\Delta B_0 = 0\,\mathrm{Hz}$. Simulated MRF signals were matched with the in vivo dictionary, and the relative percentage difference (Matching Error (ME)) was calculated. The ME is calculated as the relative deviation of an estimated X parameter from its underlying ground truth X_{true}, where

$$\mathrm{ME} = \frac{X - X_{\mathrm{true}}}{X_{\mathrm{true}}} \cdot 100\%. \tag{S.1}$$

The influence of a biased assumption on the estimates is assessed in Fig. S2. ΔB_0 matching errors (ME) are not shown, as no ME was detected. More importantly, k_{CK} estimates are only moderately affected by the bias of the fixed parameters, an $\pm 25\%$ bias range of T_1^{ATP}, T_2^{ATP}, and C_{B1} resulting in k_{CK} ME's mostly below 10%. C_{r} and

k_{CK} exceed an ME of 25% only for large bias in the assumptions ($> \pm 40\%$). C_{B1} potentially has the strongest influence on the estimates, especially for T_1^{PCr}.

Supplementary information 3: The effect of SNR on parameter estimation

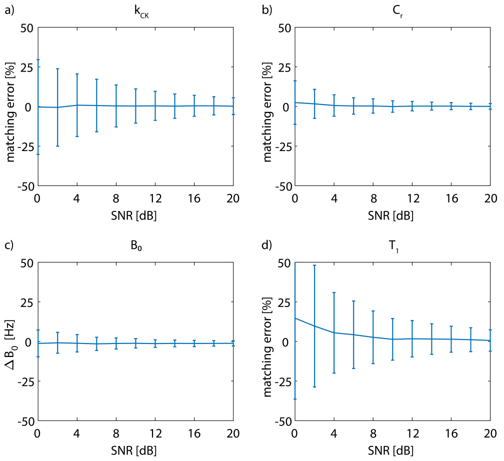

Fig. S3 Noise robustness evaluation: results of the Monte Carlo simulations displaying the matching error [%] of k_{CK}, C_{r} and T_1^{PCr} and the absolute difference for over the SNR [dB].

To evaluate the influence of noise on the parameter estimation, Monte Carlo (MC) simulations were performed. 1000 signal evolutions were simulated with free parameters set to ground-truth values according to a uniform random distribution. Parameter ranges were 0.15 to 0.45 s^{-1} for k_{CK}, 0.9 to 2 for C_{r}, -15 to 15 Hz for ΔB_0, and 4.5 to 8.5 s for T_1^{PCr}. Before phasing and matching, white Gaussian noise was added to each signal. The ME was evaluated for SNR from 0 dB to 20 dB (2 dB/step).

Fig. S3 shows the ME of the estimated parameters for different SNR, ranging from 0 dB to 20 dB. The estimates are generally not biased by the noise, except for T_1^{PCr} at low SNR ($<$ 4 dB), visible by the non-zero mean of the ME (around 20%). Standard deviations (SDs) of the MEs decrease with an increase in SNR. Overall, k_{CK}, C_{r}, and T_1^{PCr} show good noise robustness with the SD of ME below 14% for an SNR of 8 dB, 0 dB, and 10 dB, respectively. ΔB_0 (Fig. 4c) shows a SD of 1 Hz at the 4 dB SNR.

Supplementary information 4: Dictionary Quantification

Table S3 The range and steps of the dictionary were the following:

Parameter	Min	Max	Step size
k_{CK} [s^{-1}]	0.04	0.7	0.02
C_{r} [-]	0.7	0.9	0.1
	0.95	1.5	0.05
	1.6	2	0.1
	2.2	3	0.2
	3.5	6	0.5
T_1^{PCR} [s]	2	16	0.5
ΔB_0 [Hz]	-25	25	1

Supplementary information 5: Results of ROI differences

Table S5 Results of the one way ANOVA comparing ROIs with Tukey's multiple comparison test to compare all pairs of ROIs. ns = not significant; * = $p < 0.05$; ** = $p < 0.01$, *** = $p < 0.001$

	k_{CK}	Cr	T_1^{PCr}
Caudate vs Cerebellum	ns	***	ns
Caudate vs Frontal Lobe	ns	***	ns
Caudate vs Insula	ns	**	ns
Caudate vs Occipital Lobe	ns	***	ns
Caudate vs Parietal Lobe	ns	***	ns
Caudate vs Putamen	ns	ns	ns
Caudate vs Temporal Lobe	ns	***	**
Caudate vs Thalamus	ns	ns	ns
Cerebellum vs Frontal Lobe	ns	***	**
Cerebellum vs Insula	**	***	ns
Cerebellum vs Occipital Lobe	*	***	ns
Cerebellum vs Parietal Lobe	**	***	*
Cerebellum vs Putamen	ns	***	ns
Cerebellum vs Temporal Lobe	ns	ns	***
Cerebellum vs Thalamus	ns	***	ns
Frontal Lobe vs Insula	ns	*	ns
Frontal Lobe vs Occipital Lobe	ns	ns	ns
Frontal Lobe vs Parietal Lobe	ns	ns	ns
Frontal Lobe vs Putamen	ns	***	**
Frontal Lobe vs Temporal Lobe	ns	**	ns
Frontal Lobe vs Thalamus	ns	***	**
Insula vs Occipital Lobe	ns	***	ns
Insula vs Parietal Lobe	ns	*	ns
Insula vs Putamen	ns	ns	ns
Insula vs Temporal Lobe	ns	***	ns
Insula vs Thalamus	ns	***	ns
Occipital Lobe vs Parietal Lobe	ns	ns	ns
Occipital Lobe vs Putamen	ns	***	ns
Occipital Lobe vs Temporal Lobe	ns	ns	ns
Occipital Lobe vs Thalamus	ns	***	ns
Parietal Lobe vs Putamen	ns	***	**
Parietal Lobe vs Temporal Lobe	ns	**	ns
Parietal Lobe vs Thalamus	ns	***	**
Putamen vs Temporal Lobe	ns	***	***
Putamen vs Thalamus	ns	ns	ns
Temporal Lobe vs Thalamus	ns	***	***

Supplementary information 6: Results of tissue differences

Table S6 Paired t-test for Grey and White matter (Tissue) . ns = not significant; * = $p < 0.05$

	k_{CK}	Cr	T1
Tissue	ns	*	ns

Supplementary information 7: Two way ANOVA results

Table S7 Two way ANOVA test between tissue (gray and white matter) and sex. ns = not significant; * = $p < 0.05$; ** = $p < 0.01$, *** = $p < 0.001$

	Interaction tissue and sex		
Source of Variation	k_{CK}	Cr	T1
Interaction	ns	ns	ns
Tissue	ns	*	ns
Sex	ns	ns	ns

Table S8 Two way ANOVA test between ROIs and sex. ns = not significant; * = $p < 0.05$; ** = $p < 0.01$, *** = $p < 0.001$, **** = $p < 0.0001$

	Interaction ROI an sex		
Interaction	k_{CK}	Cr	T1
Interaction	ns	ns	ns
ROI	**	****	****
Sex	ns	ns	ns

Supplementary information 8: Effect of ATPase on CKI

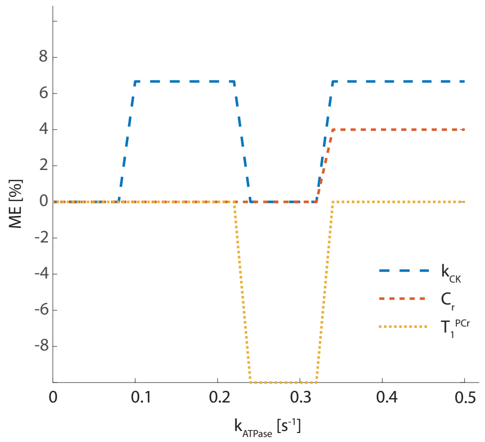

Fig. S8 Effect of the ATPase chemical exchange rate between γ-ATP and Pi (k_{ATPase}), assuming a 3 pool excahnge model, on k_{CK}, C_{r} and T_1^{PCr} estimations (2 pool model).

To investigate the effect of neglecting the third pool (Pi), connected to the γ-ATP pool via ATP synthase, simulations were conducted. The two-pool exchange model was extended to a three-pool exchange model, with the third pool, Pi, resonating at 4.82 ppm. This introduces additional parameters including the chemical exchange rate between Pi and ATP (k_{ATPase}), the longitudinal and transverse relaxation times of Pi (T_1^{Pi} and T_2^{Pi}), and the concentration ratio between Pi and ATP ($C_{\mathrm{r,Pi}}$). A total of 26 signal evolutions were simulated with k_{ATPase} ranging from 0 to 0.5 s^{-1}. The other parameters were fixed according to the closest dictionary quantification step from the results (Table 1), with $k_{\mathrm{CK}} = 0.3\ \mathrm{s}^{-1}$, $T_1^{\mathrm{PCr}} = 4.5$ s, and $C_{\mathrm{r}} = 1.25$, or to literature values: $T_1^{\mathrm{ATP}} = 0.85$ s [28], $T_2^{\mathrm{PCr}} = 135$ ms [35], $T_2^{\mathrm{ATP}} = 25$ ms [35], $T_1^{\mathrm{Pi}} = 6.7$ s [59], $T_2^{\mathrm{Pi}} = 110$ ms [60], and $C_{\mathrm{r,Pi}} = 0.28$ [59]. The signal evolutions were then matched with the dictionary used in vivo. The matching error (ME) caused by k_{ATPase} is shown in Fig. S8. A 6.5% overestimation (one quantification step) of k_{CK} can be expected within the reported literature range of k_{ATPase} (0.17 s^{-1} to 0.21 s^{-1}) [14, 42, 59].
